# Supplementary material for: Main complaints identified by parents of children with developmental delays during the initial consultation: a 10-year all-case study
Source: PeerJ. 2025 Feb 28;13:e19044. doi: 10.7717/peerj.19044 (PMC11874933; doi:10.7717/peerj.19044)
Supplement: Supplemental Information 2 [file peerj-13-19044-s002.doc]

STROBE Statement—Checklist of items that should be included in reports of ***cross-sectional studies***

|  |  | Item No | Recommendation |
| --- | --- | --- | --- |
|  | **Title and abstract〇: in title and abstract.**  **〇: Mainly described in the methods and conclusions of the abstract.** | 1 | (*a*) Indicate the study’s design with a commonly used term in the title or the abstract |
|  |  |  | (*b*) Provide in the abstract an informative and balanced summary of what was done and what was found |
|  | Introduction | | |
|  | Background/rationale  **〇: They are listed in L63-L123, covering the current situation and systems in Japan.** | 2 | Explain the scientific background and rationale for the investigation being reported |
|  | Objectives  **〇: It was listed in L124-133.** | 3 | State specific objectives, including any prespecified hypotheses |
|  | Methods | | |
|  | Study design  **〇: It was listed in L148-149.** | 4 | Present key elements of study design early in the paper |
|  | Setting  **〇: It was listed in L137-139.** | 5 | Describe the setting, locations, and relevant dates, including periods of recruitment, exposure, follow-up, and data collection |
|  | Participants  **〇: It was listed in L137-142.** | 6 | (*a*) Give the eligibility criteria, and the sources and methods of selection of participants |
|  | Variables  **〇: All outcomes are noted in the results. In this study, Age and diagnosis were treated as potential confounders.** | 7 | Clearly define all outcomes, exposures, predictors, potential confounders, and effect modifiers. Give diagnostic criteria, if applicable |
|  | Data sources/ measurement  **〇: It was listed in L153-174.** | 8* | For each variable of interest, give sources of data and details of methods of assessment (measurement). Describe comparability of assessment methods if there is more than one group |
|  | Bias  × | 9 | Describe any efforts to address potential sources of bias |
|  | Study size  ×　This is not applicable as this study is a survey of all cases. | 10 | Explain how the study size was arrived at |
|  | Quantitative variables  **〇:** | 11 | Explain how quantitative variables were handled in the analyses. If applicable, describe which groupings were chosen and why |
|  | Statistical methods  **〇: (b)**  **It was listed in L177-179.**  **(c) It was listed in L141-142.** | 12 | (*a*) Describe all statistical methods, including those used to control for confounding　× |
|  |  |  | (*b*) Describe any methods used to examine subgroups and interactions　**〇** |
|  |  |  | (*c*) Explain how missing data were addressed　**〇** |
|  |  |  | (*d*) If applicable, describe analytical methods taking account of sampling strategy　× |
|  |  |  | (*e*) Describe any sensitivity analyses　× |
|  | Results | | |
|  | Participants  **〇:** | 13* | (a) Report numbers of individuals at each stage of study—eg numbers potentially eligible, examined for eligibility, confirmed eligible, included in the study, completing follow-up, and analysed　**〇** |
|  |  |  | (b) Give reasons for non-participation at each stage　**〇** |
|  |  |  | (c) Consider use of a flow diagram　× |
|  | Descriptive data  **〇: Participant characteristics are listed in Table 2.** **Handling of missing data is described in L137-142.** | 14* | (a) Give characteristics of study participants (eg demographic, clinical, social) and information on exposures and potential confounders　× |
|  |  |  | (b) Indicate number of participants with missing data for each variable of interest　**〇** |
|  | Outcome data  **〇: Summary measures are listed in Table 2.** | 15* | Report numbers of outcome events or summary measures　**〇** |
|  | Main results  × | 16 | (*a*) Give unadjusted estimates and, if applicable, confounder-adjusted estimates and their precision (eg, 95% confidence interval). Make clear which confounders were adjusted for and why they were included　× |
|  |  |  | (*b*) Report category boundaries when continuous variables were categorized　× |
|  |  |  | (*c*) If relevant, consider translating estimates of relative risk into absolute risk for a meaningful time period　× |
|  | Other analyses  × | 17 | Report other analyses done—eg analyses of subgroups and interactions, and sensitivity analyses |
|  | Discussion | | |
|  | Key results  **〇: The information is provided at the beginning of each paragraph of the discussion. Please review the following: L213-215, L222-224, L245.** | 18 | Summarise key results with reference to study objectives |
|  | Limitations  **〇: It has been written it in L270-277 in accordance with the submission regulations.** | 19 | Discuss limitations of the study, taking into account sources of potential bias or imprecision. Discuss both direction and magnitude of any potential bias |
|  | Interpretation  **〇:** | 20 | Give a cautious overall interpretation of results considering objectives, limitations, multiplicity of analyses, results from similar studies, and other relevant evidence |
|  | Generalisability  **〇: The following section of the discussion has been filled out. Please review the following: L218-221, L261-268** | 21 | Discuss the generalisability (external validity) of the study results |
|  | Other information | | |
|  | Funding  **〇: It was wrote it according to the submission regulations.** | 22 | Give the source of funding and the role of the funders for the present study and, if applicable, for the original study on which the present article is based |

*Give information separately for exposed and unexposed groups.

**Note:** An Explanation and Elaboration article discusses each checklist item and gives methodological background and published examples of transparent reporting. The STROBE checklist is best used in conjunction with this article (freely available on the Web sites of PLoS Medicine at http://www.plosmedicine.org/, Annals of Internal Medicine at http://www.annals.org/, and Epidemiology at http://www.epidem.com/). Information on the STROBE Initiative is available at www.strobe-statement.org.
